# Fetal hypoxia secondary to severe maternal anemia as a causative link between blueberry muffin baby and erythroblastosis: a case report

**DOI:** 10.1186/s13256-016-0924-5

**Published:** 2016-06-13

**Authors:** Maria Pia De Carolis, Silvia Salvi, Iliana Bersani, Serafina Lacerenza, Costantino Romagnoli, Sara De Carolis

**Affiliations:** 1 Division of Neonatology, Department of Obstetrics, Gynaecology and Paediatrics, Catholic University of the Sacred Heart, Rome, Italy; 2 High Risk Pregnancies Unit, Department of Obstetrics, Gynaecology and Paediatrics, Catholic University of the Sacred Heart, Largo F. Vito, 1, 00168 Rome, Italy

**Keywords:** Blueberry muffin lesion, Nucleated red blood cells (NRBC), Maternal anemia

## Abstract

**Background:**

Neonatal blueberry muffin lesions are rare cutaneous eruptions, presenting as transient, non-blanching, red-violaceous papules, mostly localized in the trunk, head and neck, attributable to a marked dermal hematopoietic activity. Congenital infections of the TORCH complex (toxoplasmosis, other, rubella, cytomegalovirus and herpes) and hematological disorders have been classically associated with this neonatal dermatological manifestation. We report for the first time an unusual presentation of blueberry muffin lesions in a neonate born from a mother affected by severe anemia during pregnancy.

**Case presentation:**

A male, white Caucasian, neonate showed a cutaneous rash at birth, suggestive of “blueberry muffin”-like lesions. These cutaneous lesions were associated with marked elevation of the circulating nucleated red blood cells, and with ultrasound findings of peculiar brain ischemic porencephalic lesions. The clinical features of spontaneous disappearance and the association with marked erythroblastosis strongly suggest that these dermatological findings may be the consequence of an extramedullary hematopoiesis unexpectedly evoked by the intrauterine chronic exposure to hypoxia caused by severe maternal anemia.

**Conclusions:**

In conclusion, fetal hypoxia secondary to severe maternal anemia may play a causative and unreported role in the development of neonatal blueberry muffin lesions.

## Background

Neonatal cutaneous eruption differential diagnosis is challenging because multiple conditions may play a causative role in the pathogenesis of a cutaneous rash in a neonate such as congenital infections, hemolytic disease, twin-twin transfusion syndrome, fetomaternal hemorrhage, intracranial bleeding, and neoplastic, autoimmune or hematological disorders [1–9].

Among these cutaneous manifestations, the blueberry muffin lesions are described as a rare cutaneous eruption, presenting with transient, red-violaceous papules, attributable to a marked dermal hematopoietic activity classically explained by congenital infections and hematological diseases. An unusual presentation of blueberry muffin lesions associated with marked elevation of the circulating nucleated red blood cells (NRBC) and with ultrasound findings of brain ischemic lesions is described in a neonate born from a mother affected by severe anemia during pregnancy.

## Case presentation

A 1530-gram, male, white Caucasian, neonate (50°–75° pc) was born at 30 gestational weeks by urgent cesarean section due to an abnormal nonstress test associated with ultrasound findings of oligohydramnios and cerebral blood flow redistribution.

**Fig. 1:**
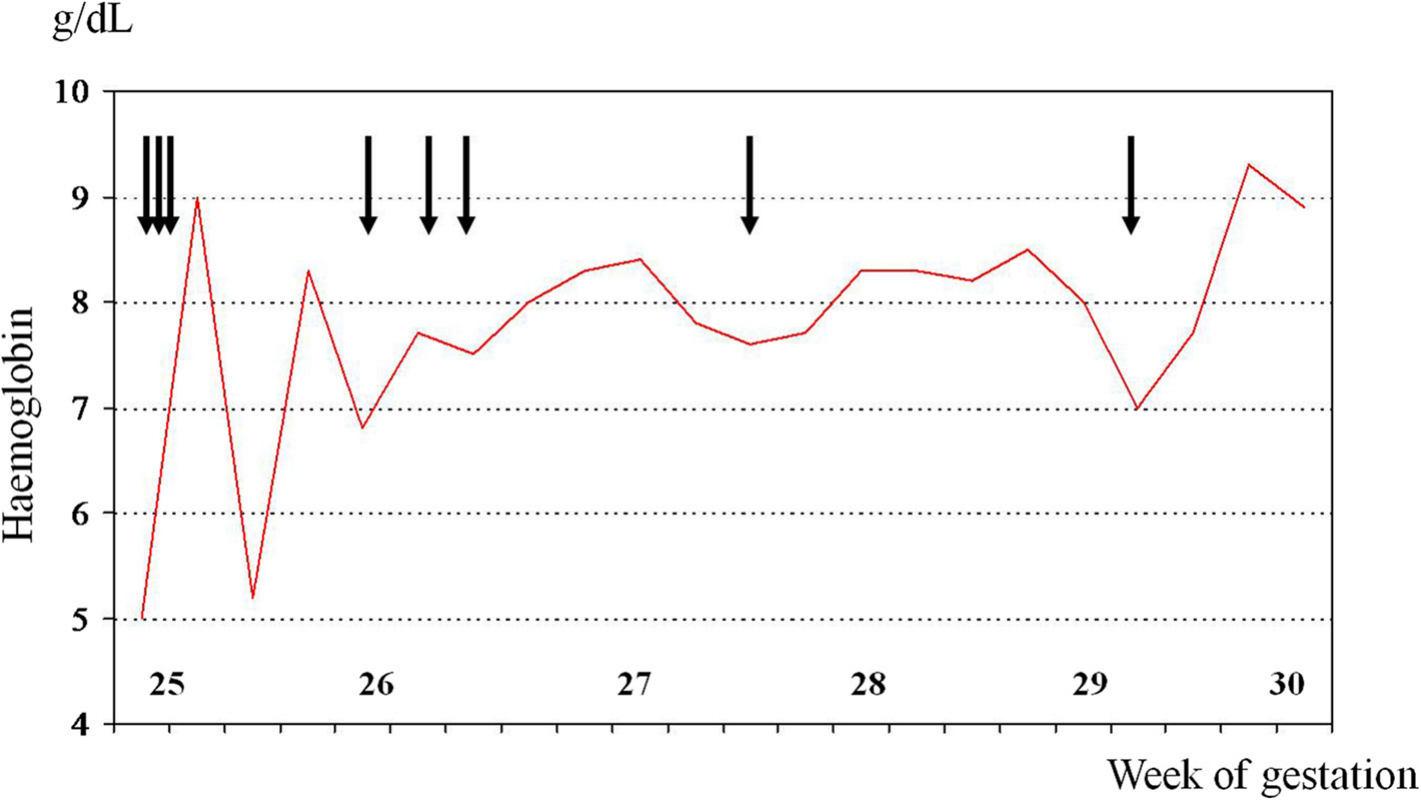
Maternal hemoglobin values and red blood cell transfusions (*black arrows*) throughout gestation

The pregnancy was uneventful until the 24^th^ gestational week, when multiple episodes of massive vaginal bleeding caused severe maternal anemia (hemoglobin 5.0 g/dL) necessitating three red blood cell (RBC) transfusions. The woman was referred to our division at 25 weeks of gestation. A voluminous and dyshomogeneous anterolateral cervical myoma was diagnosed; during the admission, repeated vaginal bleeding occurred and five other RBC transfusions were administered trying to correct the worsening maternal anemia (Fig. 1).

Maternal TORCH (toxoplasma, rubella, cytomegalovirus, herpes virus 1 and 2) screening and both antigens and antibodies for parvovirus B19, hepatitis B, hepatitis C, and syphilis were not compatible with recent infection. There were no clinical signs of chorioamnionitis. The woman was Rhesus positive and her indirect Coombs test result was negative.

The obstetric scans showed normal growth and normal uterine artery resistance with oligohydramnios and progressively deteriorating blood flow redistribution. A prenatal steroid prophylaxis was administered.

During the cesarean section, a fragmented placenta was noticed. The histopathological examination of the placenta showed signs of chronic hypoxia exposure, as there was terminal villous hypotrophy, infarctual areas, and intervillous fibrinoid deposition.

**Fig. 2:**
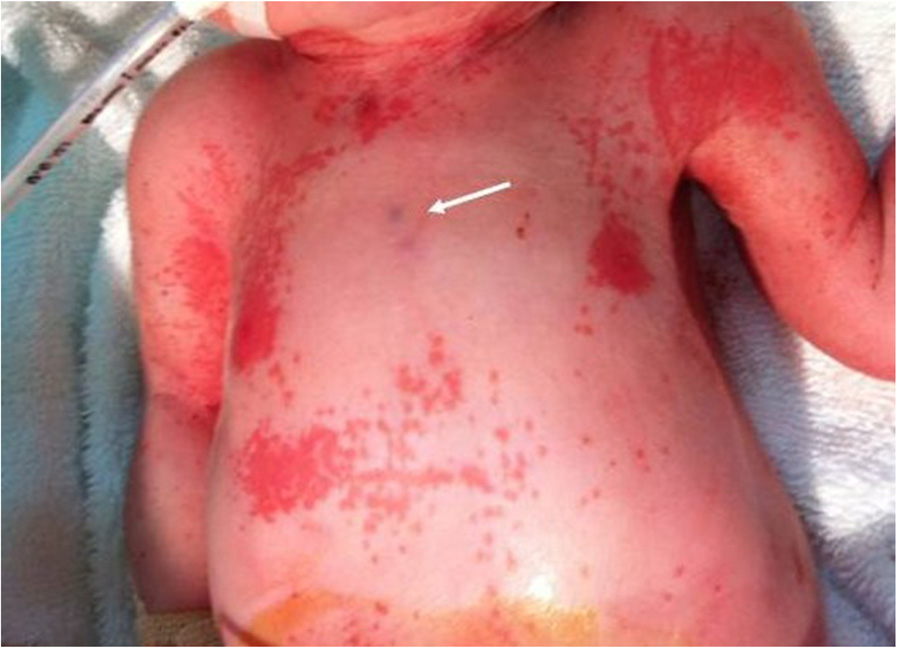
Skin eruption at birth: a red and violaceous (*arrow*), non-blanching macular rash localized at head, neck, and trunk

At the birth, the neonate exhibited a severe cardiorespiratory depression (Apgar score: 1^1’^- 4^5’^- 7^10’^- 8^15’^- 8^20’^) requiring ventilation and oxygen [fraction of inspired oxygen (FiO_2_) 100 %]. His umbilical pH and base excess levels were 6.97 and -12.2 mmol/L respectively, indicating perinatal acute asphyxia. On physical examination, he showed a skin eruption consisting of a red-violaceous, non-blanching macular rash mostly localized at his head, neck and trunk (Fig. 2).

During the first days of life, he developed severe multiorgan postasphyxial syndrome involving his brain, heart, liver, kidneys, and lungs, and complicated by disseminated intravascular coagulation.

**Fig. 3:**
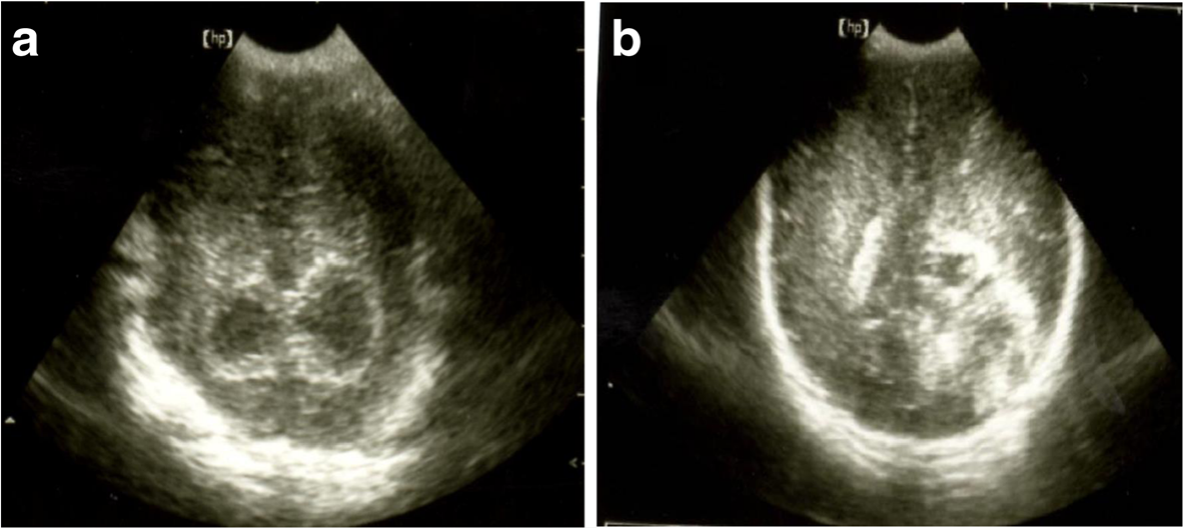
Bilateral subcortical, hypoechoic, porencephalic lesions in the posterior temporal region (**a**); periventricular hyperechogenicities (**b**)

On day of life (DOL) 1 the neonate, while still being ventilated, showed increased level of alanine aminotransferase (104 IU/L, normal range 14–70 IU/L), hypoalbuminemia (2.4 g/dL, normal range 3.8–5.4 g/dL), and cardiac troponin T value equal to 0.155 μg/L (normal range 0.02–0.13 μg/L). An echocardiographic evaluation showed marked distension of the right sections associated with a moderately hypokinetic right ventricle and moderate tricuspid insufficiency; a patent ductus arteriosus with bidirectional shunt and an ejection fraction equal to 76 % were also detected. His blood type was Rhesus positive and his direct Coombs test result was negative. A hemocromocytometric examination revealed normal hemoglobin and hematocrit values, but low platelet count levels with an apparently marked leukocytosis due to the presence of a high rate of NRBC and high reticulocytes count (Table 1). Abnormal coagulation test results were also recorded (Table 1). Platelet and frozen plasma transfusions were consequently administered (10 mL/kg). C-reactive protein, TORCH screening, and tests for both antigens and antibodies for parvovirus B19, adenovirus, hepatitis B, hepatitis C, Coxsackie virus, Epstein-Barr virus, and syphilis were negative. The thrombophilia screening was negative. A brain ultrasound scan revealed brain edema, periventricular hyperecogenicities and bilateral, hypoechoic, porencephalic lesions in the subcortical posterior regions (Fig. 3).

**Table 1:** Laboratory data of the neonate at various time points

	DOL 1	DOL 2	DOL 3	DOL 7	DOL 28
WBC (× 10^9^/L)	23,540	20,040	19,600	17,530	13,000
NRBC/100 WBC	270	253	200	4	0
Absolute NRBC/mm^3^	63,558	50,701	39,200	701	0
Reticulocytes (× 10^9^/L)	519	394	342	90	31
PLT (× 10^9^/L)	38	58	137	194	339
Hematocrit (%)	54.9	50	49.5	43.5	31.9
Hemoglobin (g/dL)	16.1	15.8	15.2	14.4	10.2
PT (s)	>2 minutes	20.6	18.2	18.0	11.3
aPPT (s)	56.0	40.1	36.3	30.3	29.9
Fibrinogen (g/L)	n.d.	69	187	191	233
D-dimers (ng/mL)	9419	2728	957	888	322

*DOL* day of life, *WBC* white blood cell, *NRBC* nucleated red blood cell, *PLT* platelet count, *PT* prothrombin time, *aPTT* partial thromboplastin time, *n.d.* not detectable

On DOL 2, blood tests highlighted high level of creatinine (1.7 mg/dL, normal value <1.5 mg/dL), with persistently abnormal coagulation test results and thrombocytopenia (Table 1), so further platelet (10 mL/kg) and frozen plasma transfusions (10 mL/kg) were administered.

On DOL 4, mechanical ventilation was stopped and the neonate was switched to nasal continuous positive airway pressure (CPAP), renal and hepatic dysfunction was improved, and his platelet count and coagulation tests were normalized. On the same day, his cutaneous rash was also attenuated.

On DOL 7, CPAP was discontinued and there was complete resolution of the skin eruption.

Serial brain ultrasound evaluations performed during the first week of his life showed a progressive normalization of parenchymal echogenicity but persistence of the bilateral porencephalic areas in the temporal and occipital regions. Brain magnetic resonance imaging, performed on DOL 10, confirmed the presence of porencephalic areas in the parietal and occipital regions on the right, and in the parietal, temporal, and occipital regions on the left.

The neonate was discharged on DOL 41. He had been growing normally and his neurologic examination showed mild hypotonia. A visit at 3 months of age showed no neurologic impairment.

## Discussion

In this report, an unreported presentation of blueberry muffin-like lesions in a neonate born from a mother who had severe and long-lasting anemia during pregnancy is presented.

Neonatal cutaneous eruption differential diagnosis is challenging because multiple conditions have to been included: congenital infections, chronic prenatal anemia caused by hemolytic disease (Rh-ABO-Kell incompatibility), hereditary spherocytosis, twin-twin transfusion syndrome, fetomaternal hemorrhage and severe intracranial bleeding, neoplastic disease (neuroblastoma, histiocytosis, leukemia, and rhabdomyosarcoma), neurodegenerative disease (Aicardi-Goutières syndrome), multiple hemangiomas, multifocal lymphangioendotheliomatosis, blue rubber bleb nevus syndrome, multiple glomangiomas, and systemic lupus erythematosus [1–9]. However, in the present case, none of the known mentioned causative factors was present. The only significant feature detected at the birth was the extremely marked erythroblastosis, even much higher than usually described in cases of perinatal asphyxia [10]. The hypoxic stimuli of perinatal acute asphyxia may be able to stimulate erythropoietin (EPO) production, resulting in an exaggerated erythropoiesis, with the release of immature NRBC into the fetal circulation. Nevertheless, according to the literature, the magnitude of the circulating NRBC count is usually much lower than that found in our case [10].

The extremely high NRBC count found in our neonate should be not only the result of the perinatal asphyxia, but mostly of the chronically repeated hypoxic injuries due to maternal anemia. The worsening maternal anemia secondary to recurrent vaginal bleeding (hemoglobin values <6 g/L at least twice during pregnancy) required multiple administrations of RBC transfusions: this maternal treatment may have created a fluctuating hypoxic state in the fetus, resulting in additional and sequential stimuli for EPO production and consequent NRBC release. Confirming these observations, two cases of blueberry muffin lesions following recombinant EPO administration in premature neonates have recently been reported [11, 12].

Detectable signs of this chronic hypoxic damage are both the histopathological placental findings and the detectable neonatal brain ischemic lesions already observed on DOL 1. Contrariwise clinical signs of the acute perinatal asphyxia were the multiorgan postasphyxial syndrome and the diffuse brain edema that progressively improved during the first days of life.

The clinical features of the cutaneous rash such as its clinical appearance, macular-like, red-violaceous, mostly involving head, neck and trunk lesions, its gradual disappearance within DOL 8, and its association with marked erythroblastosis and reticulocitosis, led us to consider these skin lesions as compatible with a diagnosis of blueberry muffin lesions secondary to extramedullary hematopoiesis. All these clinical aspects were more suggestive of blueberry muffin-like lesions despite the laboratory evidence of an abnormal coagulation test and thrombocytopenia. A blueberry muffin lesion is typically non-blanching, red-violaceous macules or firm, dome-shaped papules (2–8 mm in diameter), usually generalized but more pronounced in the head, neck and trunk. New lesions seldom appear after birth while resolution of the skin disorders usually occurs by the first 3–6 weeks of life.

During normal embryologic development, extramedullary hematopoiesis occurs in different organs including the dermis. Nevertheless, the erythroblastic elements are normally phagocytized by leukocytes by the last period of gestation [13]. In cases of marked dermal hematopoietic activity, cutaneous lesions may also persist after the birth, resulting in purpuric lesions defined as blueberry muffin lesions. Unfortunately, as in other similar cases reported in the literature [3], it was not possible to perform a biopsy of the skin lesions due to the neonate’s critical clinical condition during the first days of life and to their relatively rapid disappearance.

## Conclusions

The peculiarity of the present case is that the severe and long-lasting maternal anemia, requiring eight RBC transfusions and responsible for fetal chronic hypoxia, is the only causative factor responsible for the marked elevation of NRBC count and probably for the blueberry muffin-like cutaneous lesions appearance. In conclusion, we suggest that in blueberry muffin babies, fetal hypoxia secondary to severe maternal anemia may play a causative and unreported role in the development of these cutaneous lesions, and therefore has to been considered in the differential diagnosis in addition of all precedently known causes.

## Abbreviations

CPAP: continuous positive airway pressure
EPO: erythropoietin
DOL: day of life
NRBC: nucleated red blood cells
RBC: red blood cell
